# Thin Film Multi-Electrode Softening Cuffs for Selective Neuromodulation

**DOI:** 10.1038/s41598-018-34566-6

**Published:** 2018-11-06

**Authors:** María A. González-González, Aswini Kanneganti, Alexandra Joshi-Imre, Ana G. Hernandez-Reynoso, Geetanjali Bendale, Romil Modi, Melanie Ecker, Ali Khurram, Stuart F. Cogan, Walter E. Voit, Mario I. Romero-Ortega

**Affiliations:** 1 Department of Bioengineering, University of Texas at Dallas, 800 W. Campbell Road, Richardson, TX 75080 USA; 2 Department of Material Science and Engineering, University of Texas at Dallas, 800 W. Campbell Road, Richardson, TX 75080 USA

## Abstract

Silicone nerve cuff electrodes are commonly implanted on relatively large and accessible somatic nerves as peripheral neural interfaces. While these cuff electrodes are soft (1–50 MPa), their self-closing mechanism requires of thick walls (200–600 µm), which in turn contribute to fibrotic tissue growth around and inside the device, compromising the neural interface. We report the use of thiol-ene/acrylate shape memory polymer (SMP) for the fabrication of thin film multi-electrode softening cuffs (MSC). We fabricated multi-size MSC with eight titanium nitride (TiN) electrodes ranging from 1.35 to 13.95 × 10^−4^ cm^2^ (1–3 kΩ) and eight smaller gold (Au) electrodes (3.3 × 10^−5^ cm^2^; 750 kΩ), that soften at physiological conditions to a modulus of 550 MPa. While the SMP material is not as soft as silicone, the flexural forces of the SMP cuff are about 70–700 times lower in the MSC devices due to the 30 μm thick film compared to the 600 μm thick walls of the silicone cuffs. We demonstrated the efficacy of the MSC to record neural signals from rat sciatic and pelvic nerves (1000 µm and 200 µm diameter, respectively), and the selective fascicular stimulation by current steering. When implanted side-by-side and histologically compared 30 days thereafter, the MSC devices showed significantly less inflammation, indicated by a 70–80% reduction in ED1 positive macrophages, and 54–56% less fibrotic vimentin immunoreactivity. Together, the data supports the use of MSC as compliant and adaptable technology for the interfacing of somatic and autonomic peripheral nerves.

## Introduction

Peripheral nerve interfaces (PNIs) connect the human peripheral nervous system to electronic devices most frequently to facilitate functional electrical stimulation in patients with some level of disability^1,2^. Current PNIs may be categorized based on their fabrication, sensitivity and invasiveness^3,4^. Cuff electrodes are moderately invasive PNIs implanted circumferentially on the peripheral nerves, and made of flexible materials with helical, spiral, split-cylinder or folding designs to conform to their cylindrical shape^5,6^. Traditional cuff electrodes fabricated in silicone are commonly used due to their softness (1–50 MPa) and chronic stability, although their fabrication is mostly limited to molding and lamination techniques^1,7–9^. Unfortunately, these cuffs often evoke a significant foreign body immune response including epineural fibrosis which restricts nerve stretching, compromising nerve conduction^10^, and negatively affecting the sensitivity and stimulation thresholds of the electrodes^11–13^. In addition, cuff thickness (200–600 μm), sharp edges and inadequate cuff-nerve fitting, further exacerbate this fibrotic response^1,14^.

Multi-contact cuffs are often used for selective recording and stimulation from individual nerve fascicles innervating different muscle targets^15–17^. However, current methods and materials for high precision manufacturing of multi-contact cuff electrodes have critical limitations^18^. High-resolution photolithographic fabrication of thin and flexible electrodes using ribbon-like materials such as polyimide have been reported^19^, yet this polymer is relatively rigid (2.5 GPa), and cuffs made of this material bear the risk of nerve damage and inflammation. This has motivated the development of hydrogel and nanofiber coatings onto polyimides to provide a softer interface, which complicates device manufacturing^20,21^.

We previously suggested the use of thiol-ene/acrylate based shape memory polymers (SMP) for neural interfaces, as they can be photolithographically processed in thin films (5–50 µm) for the fabrication of neural devices designed for various targets, including cortical probes, spinal cord stimulators, and nerve cuffs^22–25^. Early SMP formulations were shown to soften from 1,809 MPa at room temperature to 41 MPa after 20 min at 37 °C, with minimal water uptake (1.11% by volume)^26,27^. This first iteration of the SMP cuff was pre-programmed for self-wrapping around the rat vagus nerve, and was capable of evoking bradycardia acutely after electrical stimulation using Au electrodes^26,28^. However, the cuff was only able to curl around the nerve like a hook due to its limited curvature, making only partial contact with the tissue. Here we report the use of a new generation of thiol-ene/acrylate for the manufacture of multi-electrode nerve cuff devices. Several designs of Au and titanium nitride (TiN) multi-electrodes were fabricated and tested to show electrochemical performance and functionality *in vitro*, and to demonstrate acute and sub-chronic recording and stimulation of the rat sciatic and pelvic nerves (1–1.5 mm and 200 µm diameter, respectively). These multi-electrode softening cuff (MSC) devices were also used to evoke monopolar and bipolar selective stimulation of the gastrocnemius and tibialis anterior muscles in the hind limb, from a cuff implanted in the sciatic nerve. Finally we provide immunohistochemical evidence of reduced foreign body response by the SMP devices, compared to silicone nerve cuffs implanted side-by-side in the rat sciatic nerve for 30 days.

## Materials and Methods

### Design and fabrication of multi-size softening cuff electrodes

Three SMP devices were manufactured: One with 12-contacts (MSC-12) that fits a 1000 µm diameter nerve, and two with 4 or 16 contacts (MSC-4 and MSC-16) to fit nerves ranging from 100–1000 µm in diameter (Table 1). The electrodes were fabricated using traditional photolithographic techniques, with Au and TiN electrode contacts to increase the charge injection and storage capacity as reported elsewhere^29,30^. These devices were closed either with a suture through a single eyelet for anchoring the distal end to the underlying muscle (MSC-4 and MSC-16), or through two pairs of eyelets aligned after rolling the SMP ribbon into a cylindrical cuff (MSC-12).

**Table 1:** Multi-electrode softening cuffs (MSC) designs.

Cuff electrode design	Nerve diameters adapting	Number of contacts	Conductive materials	Closure mechanisms
MSC-4	100–1000 µm	4	TiN	Suture and silicone elastomer
MSC-12	1000 µm	12	TiN	Suture
MSC-16	100–1000 µm	16	TiN and Au	Suture

Three cuff electrode designs with variations in number and material of electrode contacts, closure mechanisms and target nerve diameters.

**Figure 1:**
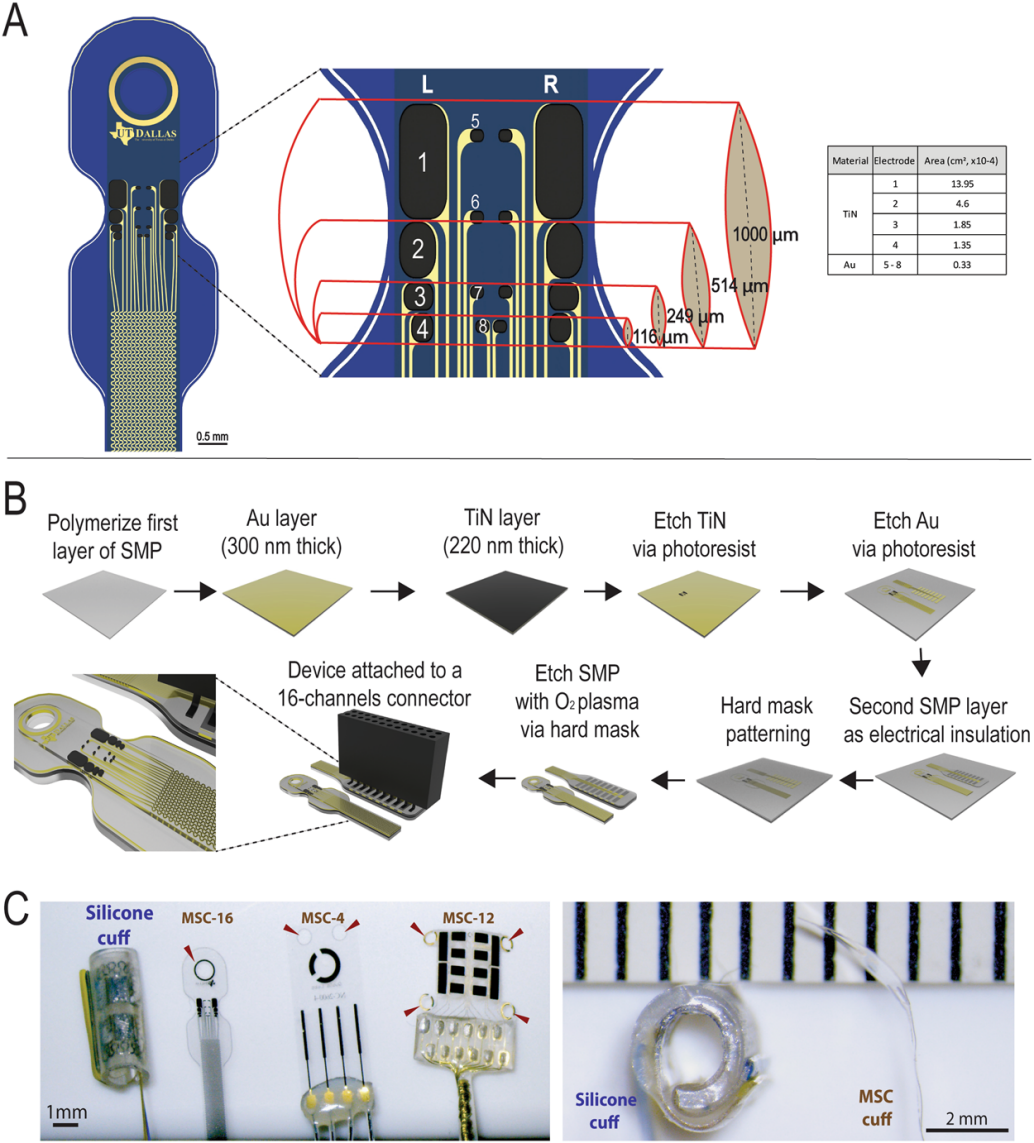
Design and fabrication of MSC devices. (**A**) Schematic of the MSC-16 illustrating the TiN (1–4) and Au (5–8) electrodes in rows of different sizes aligned in right and left columns (R and L), designed to fit on nerves of different diameters (from 100 to 1000 µm). The table list the surface geometric area of the electrodes. (**B**) Fabrication process: thiol-ene/acrylate thin films were used for the transference of Au. The TiN was sputtered and etched. (**C**) Photographs of the silicone and different designs of MSC cuffs used in the study, the arrows indicate the eyelids included for suture (left). Cross section view of silicone cuff with thick walls, compared to the thin MSC device (right). Ruler in C shows the scale in mm.

Figure 1A shows the MSC-16 design which consist of eight TiN electrodes organized in two columns and four rows in gradually increasing electrode size (0.1 to 0.5 mm long) resulting in geometric surface area of 1.35, 1.85, 4.60, and 13.95 × 10^−4^ cm^2^. The TiN electrodes flanked two central columns of same size Au electrodes (0.046 mm long; 3.3 × 10^−5^ cm^2^), 2 per row.

The synthesis and characterization of the SMP have been previously described^22^. In brief, a 300 nm of Au layer was deposited using electron-beam evaporation on clean glass slides. The SMP was synthesized by mixing stoichiometric quantities of the monomers 1,3,5-triallyl-1,3,5-triazine-2,4,6-trione and tris [2-(3-mercaptopropionyloxy)ethyl] isocyanurate with 31 mol% tricyclodecane dimethanol diacrylate and 0.1 wt% of 2,2-dimethoxy-2-phenylacetophenone as photoinitiator, cast between two glass slides and cured in 365 nm UV oven for 1 h and 120 °C vacuum oven for 12 h. The glass slides were then separated, resulting in the Au film transferring onto the SMP^26^. A 220 nm thick TiN layer was deposited over the Au by RF sputtering. The TiN and the Au wires were then patterned using standard photolithography and wet etching methods. A second layer of SMP was deposited by spin coating, and photo-polymerized using UV and vacuum ovens. The first and second layers of SMP served as electrical insulation in the device, encapsulating the Au wires (Fig. 1B). The superficial topography of the TiN coated electrodes patterned onto the SMP was evaluated using SEM Supra-40, Zeiss microscope at 5.0 kV. Devices were then packaged with 18-channel nano Omnetics® connectors using a solder reflow process and sealed by applying additional SMP. Two extra stainless-steel wires were bonded to a 18-channel Omnetics connector to serve as the reference and ground. These devices were mounted on pedestals with metallic caps to implant in the back of the rat for the sub-chronic experiments. Figure 1C shows photographs of the MSC devices used in this study compared to a commercial silicone cuff electrode.

### Dynamic mechanical analysis (DMA)

To evaluate the mechanical properties of the SMP substrate, a solid mechanical analyzer (RSA-G2, TA Instruments) was used. The storage modulus (E′) and tan δ were measured in air (dry) and in phosphate buffered saline (PBS). Measurements were performed on rectangular samples of SMP (4.5 ± 0.1 × 45 ± 3 mm; 30 μm thick), using a 15 mm clamping distance, a 0.2 N preload force at 1 Hz with deformation amplitude of 0.275% strain. Dry experiments were run from 10 to 120 °C using a heating rate of 2 °C min^−1^. Soaking experiments were performed using the immersion system of the RSA-G2 filled with PBS and done isothermally for 60 minutes at 37 °C, followed by cooling at 3 °C min^−1^ and subsequent heating from 10 to 85 °C at 2 °C min^−1^. An offset of about 10 °C between the temperature sensor outside the immersion chamber responsible for temperature controlling and the actual temperature inside the immersion bath was considered for the graphic representation of the temperature of the polymer sample inside the solution.

### *In vitro* and *in vivo* electrochemical characterization

*In vitro* electrochemical impedance spectroscopy (EIS) and cyclic voltammetry (CV) was used to evaluate each electrode in the MSC devices using a Gamry® Reference-600 potentiostat in a three-electrode configuration. A Pt wire counter electrode and a Ag|AgCl reference electrode were used in air equilibrated PBS (pH = 7.2) at room temperature. The EIS measurements were made between 1 Hz and 100 kHz by applying a 10 mV RMS sinusoidal signal on top of the resting potential of each electrode. CVs were performed between −0.6 V and 0.8 V at a sweep rate of 50 mV/s for both TiN and Au electrodes. Cathodic charge storage capacity (CSC) was calculated from the time integral of the cathodic current^31^. Voltage transient measurements were obtained by using a custom-built instrument (Sigenics Inc, IL) that generates monophasic cathodal current pulses followed by potential-controlled anodic recharge phase and using a Tektronix oscilloscope for recording the electrode potential and current. All measurements employed 200 µs pulses at 50 Hz. The current in the pulse was gradually increased until the electrode potential reached −0.6 V in order to calculate the maximum charge injection capacity of the TiN electrodes. The *in vivo* electrochemical characterization was also evaluated by CV and EIS from every channel in the MSC-16 implanted in the ScN, using a Pt wire counter electrode inserted near the incision and a stainless steel wire inserted in the tail as a reference electrode.

### Animals

Thirteen adult female Lewis rats (300–350 g; Charles River, Wilmington, MA) were used for the experiments. For acute recording and stimulation studies, electrodes were implanted on the sciatic nerve (ScN; 1–1.5 mm diameter) that innervates the hind limb (n = 4), and the pelvic nerve (PN; ~200 µm diameter) that contains autonomic fibers from the bladder (n = 3). For the sub-chronic studies, rats were implanted with both a commercial pre-sized silicone cuff (1.4 mm I.D., Cortec®; Freiburg) and a MSC electrode in the ScN (n = 6). The size of the silicone cuff was considered appropriate for the ScN nerves given the size of the rats (10-11 months of age).

### Ethics statement

All protocols and surgical procedures were approved by The University of Texas at Dallas, Institutional Animal Care and Use Committee (IACUC, protocol No.14-09), following the guidelines provided by the National Institute of Health (NIH).

### Surgical procedures

Animals were implanted with electrodes on the sciatic nerve, as reported previously^32,33^. Briefly, the animals were anesthetized with vaporized isoflurane (2%) in a constant oxygen flux (2 L/min). For the ScN, a 4 cm incision was made in the hind limb below the femur and the biceps femoris and vastus lateralis muscles were separated. The connective tissue was cleared and the nerve lifted slightly to place the SMP and/or the silicone cuff electrodes for the acute or sub-chronic experiments. The SMP devices softened when placed on the tissue and subsequently folded around the nerves and either closed by suturing (9-0 USP polyimide monofilament) to the muscle using suturing holes in the devices (n = 10), or by applying medical grade silicone elastomer (Sylgard®) (n = 3).

For the PN, a midline incision was made 3 cm from the pubic bone towards the mid ventral area. The bladder and urethra were used as anatomical references to locate the PN onto which the SMP cuffs were implanted. To evoke a neural response, the bladder was filled with saline using a 25-gauge catheter inserted at the dome, infused at 300 µL/min using an automated pump (New Era Pump Systems, Inc.). The bladder pressure was monitored with a transducer (Neurolog Systems) and synchronized to the neural recording using the Omniplex Neural Data Acquisition System (Plexon Inc.). At the end of the study, lidocaine was added over the PN to block nerve activity to confirm the neural nature of the recorded signals.

### Acute and sub-chronic recordings of evoked action potentials

To demonstrate the MSC cuffs capability to record compound nerve action potentials (CNAP), the MSC-16 was implanted into the ScN, where it was used to record the evoked potential elicited by electrical stimulation using a bipolar CorTec® silicone cuff electrode 4 mm proximally. The evoked activity was recorded using the OmniPlex Neural Data Acquisition system (40 KHz sampling rate) with custom built 16 channel connector and a G2 headstage amplifier of 10 MΩ input impedance at 1 Hz, and a channel splitter to record from every electrode. A current-controlled stimulator (A-M Systems®) was used to evoke CNAP using 2 mA (approximately 3X the threshold current) at 2 Hz and 300 µs square wave symmetrical biphasic pulses, with no interphase delay. Measurements were obtained at implantation and 30 days after (sub-chronic). A stainless-steel needle electrode in the tail served as ground. Recorded CNAPs were processed offline using Offline Sorter (Plexon Inc.) with a 4-pole high-pass Butterworth filter and cut-off frequency of 250 Hz. Evoked CNAPs were detected using the threshold-crossing method and averaged throughout the stimulation window. A custom MATLAB script was used to perform spike-triggered averaging of the CNAPs. Conduction velocities of Aγ, B and C fiber types were calculated by dividing the distance between the stimulating and recording electrodes, by their respective peak latency time.

### Fascicle-specific stimulation

To demonstrate *in vivo* selective stimulation, we implanted the MSC-16 in the ScN and evoked muscle responses by using cathodic first, symmetrical biphasic 300 µs pulses, with 5 µs interphase delay delivered at 2 Hz using a PlexStim2 (Plexon Inc.) instrument with a custom-made connector; in a range from 38 to 100 µA. Current was delivered in monopolar configuration using a Pt wire return electrode in the rat tail and also in bipolar configuration using different pairs of electrodes on the MSC-16 array. Thresholds were detected by gradually increasing the current to levels at which a visible hind-limb motor recruitment was observed.

### 3D Tracking of evoked movements

The range and direction of hind limb movements evoked by stimulation of the ScN using the MSC was evaluated by 3D tracking using two video cameras (Stingray, Allied Vision Technologies®; 80 frames per second) connected to the Omniplex and Cineplex Behavioral Research Systems (Plexon, Inc.). The cameras were calibrated using a grid with 10 mm black and white squares. The ankle and toes were marked with different colors using non-toxic dyes to track the x, y, and z coordinates of the centroid of each color as a function of time. The magnitude of movement was calculated using the Euclidian distance with respect to the baseline. The movement angle was calculated from equation (1):

1 $$\theta =arcsin(\frac{\mathop{P}\limits^{\rightharpoonup }\cdot \mathop{Q}\limits^{\rightharpoonup }}{|\mathop{P}\limits^{\rightharpoonup }|\,|\mathop{Q}\limits^{\rightharpoonup }|})$$

where $\mathop{P}\limits^{\rightharpoonup }=({x}_{B},\,{y}_{B},\,{z}_{B})$ is the labeled toe at baseline, and $\mathop{Q}\limits^{\rightharpoonup }=({x}_{\bar{P}},\,{y}_{\bar{P}},\,{z}_{\bar{P}})$ is the location average-peak-twitch; the ankle marker was taken as the origin of both vectors.

### Histological analysis

The segments of the ScN implanted with silicone and MSC electrodes were isolated 30 days after implantation and histologically evaluated using immunofluorescence. The tissue was rinsed in phosphate-buffer saline (PBS), fixed in cold 4% paraformaldehyde in PBS (pH 7.2) for 24 h before cryoprotecting in cold graded sucrose solutions (10, 20, and 30% in PBS). The tissue was embedded in OCT media, cross sections cut at 35 µm in a cryostat and mounted on glass slides. For immunohistochemistry, the tissue sections were rinsed and incubated with primary antibodies: 200 kDa neurofilament axonal marker (NF-200; Sigma, N4142), myelin glycoprotein zero, (P0; Millipore, AB9352), the fibrosis marker vimentin (abcam, ab20346), and the 110 kDa activated macrophages glycoprotein maker ED1 (abcam, 31630). Secondary antibodies coupled to Alexa Fluor 488 or 555 (Invitrogen; 1:200 dilution) or Cy5 bis-NHS ester (Jackson Immunoresearch; 1:400 dilution) were used for visualization. Cell nuclei were labeled with 4′ 6-diamidino-2-phenylindole (DAPI; 0.01 mg/mL). The sections were then mounted on glass coverslips and imaged in a confocal microscope (Nikon, eclipse Ti®) at 20 and 40X magnification. Optical sections from tissue implanted with both MSC’s and silicone cuffs (8–10 µm) were reconstructed into Z-stacks using ImageJ (1.51w version). Area of fibrotic tissue around the nerves implanted with silicone or MSC electrodes was quantified, and the number of activated macrophages (ED1+ cells) measured from three representative fields per sample.

### Statistics

We used the RStudio 1.0.136 software for statistical analysis and the Levene’s test to assess equality of variance in peak amplitude (mV) by fiber type between recording materials (TiN vs. Au), followed by an unpaired two-tailed Student’s t-test between the two materials, with m = 0, confidence interval = 0.95. Conduction velocities between different electrode positions in the device by fiber type were evaluated using Analysis of Variance (ANOVA) followed by post-hoc Tukey’s test. For selective stimulation studies the charge injection was represented as the mean ± standard deviation (SD). For fibrosis, we measured the vimentin+ areas around the nerves (NF-200+) and for activated macrophages, we calculated the percentage of ED1+ cells with respect to the total cell number based on DAPI counterstaining, evaluated by two-tailed Student’s t-test, and reported as mean ± SD. P values < 0.05 were considered significant.

## Results

### *In vitro* electrochemical and mechanical characterization of MSCs

**Figure 2:**
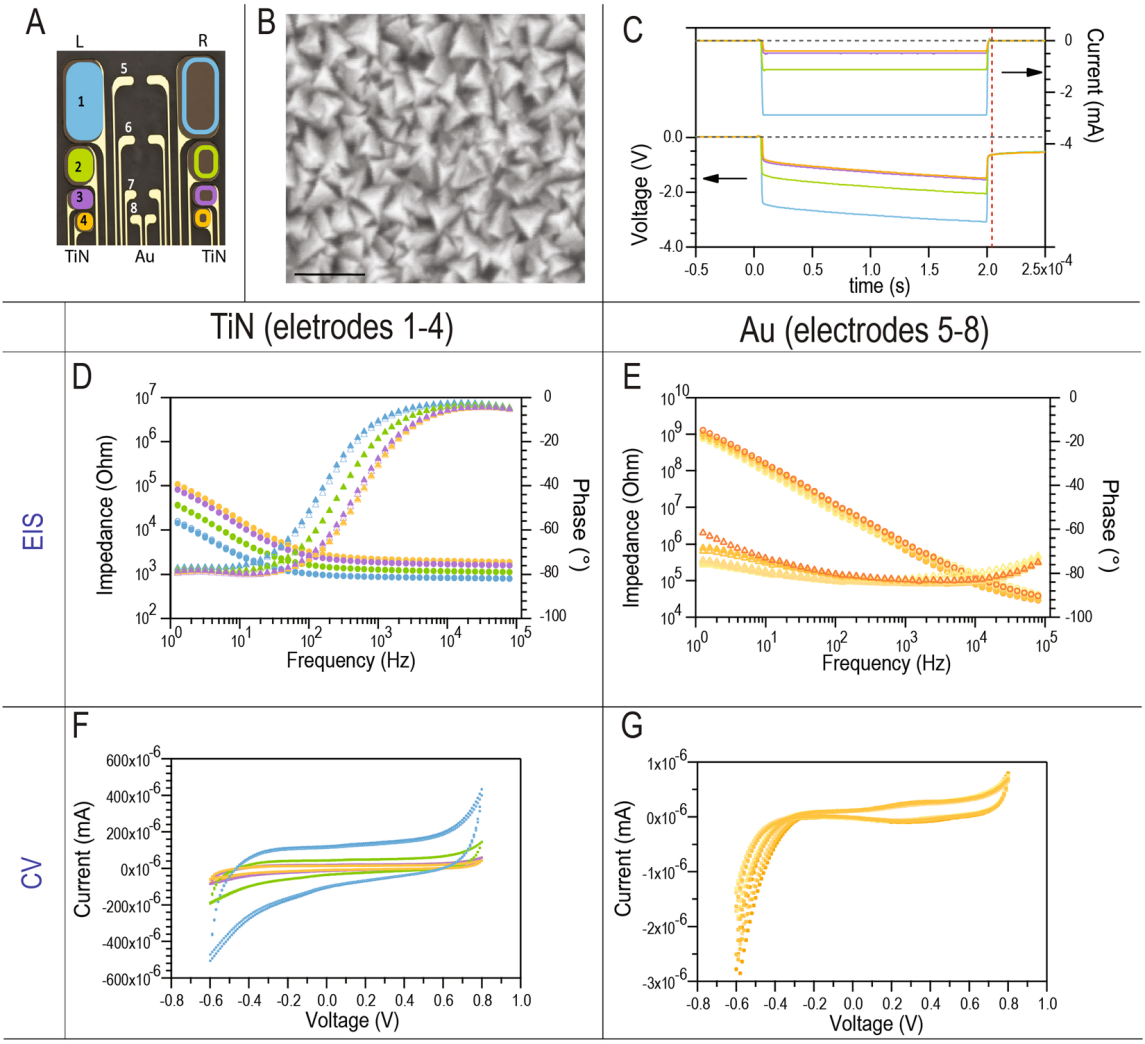
*In vitro* electrochemical characterization. (**A**) Schematic of the MSC-16 design, color-coded to correlate with the graphs, filled and open symbols represent electrodes in the left, and right side, respectively. (**B**) SEM of the pyramidal topography of the TiN coating, scale bar = 100 nm. (**C**) Voltage transients of the TiN electrodes of different sizes. Dotted lines indicate the zero values. (**D**,**E**) EIS measurements of the TiN and Au electrodes. Circles represent the impedance and triangles the phase. (**F**,**G**) shows cyclic voltammetry measurements, voltage represents the electrode potential versus a Ag/AgCl reference electrode. Top and bottom arrows in C indicate that measurements in current and voltage, respectively.

We performed electrochemical analysis for the MSC-16 electrode, and measured impedance and CSC for each of the Au and TiN contacts (Fig. 2A). The SEM evaluation of the TiN electrodes confirmed the sub-100 nm polygonal topography of this coating which increased the geometric surface area of the coated electrodes (Fig. 2B). Voltage transient measurements for the TiN contacts showed injectable currents ranging from 400 µA to 3 mA depending on the size of the electrode at maximum cathodic excursion potential of −600 mV (E_mc_; Fig. 2C). Considering the 200 µs pulse width, these values correspond to an average charge injection capacity of 500 µC/cm^2^ and 100–600 nC/phase within the water window. The 200 nm thick TiN coating, despite being thinner than those used in prior studies (10 µm)^34^, was efficient in reducing electrode impedance to an average of 1 to 3 kΩ at 1 kHz (Fig. 2D). The impedance of the Au contacts was approximately 750 kΩ at 1 kHz (Fig. 2E). Average cathodic charge storage capacity of the TiN and Au electrodes calculated from the CVs was 3 mC/cm^2^ and 0.1 mC/cm^2^, respectively (Fig. 2F,G).

**Figure 3:**
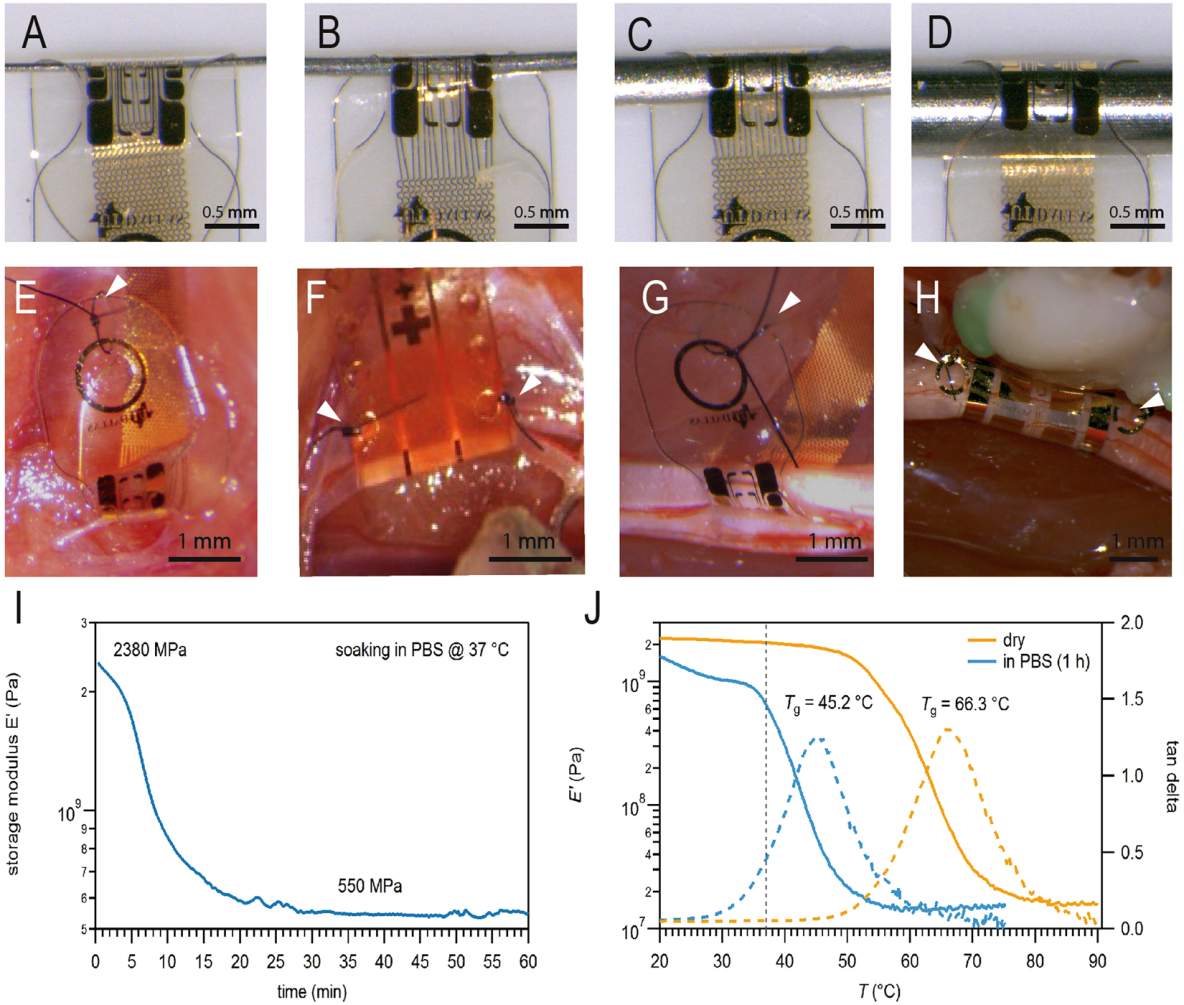
Multi-size adaptability and mechanical characterization. (**A**–**D**) The MSC-16 cuff adapts to metal rods of different diameters (100–1000 µm). SMP cuffs implanted snugly onto nerves of different diameters: (**E**) PN (200 µm), (**F**) vagus nerve (600 µm), (**G**) CPN (800 µm), and (**H**) ScN (1000 µm). Arrowheads in (**E**–**H**) point to the sutures used as cuff closing mechanism. (**I**) Dynamic mechanical analysis shows the SMP storage modulus over time in PBS at 37 °C. (**J**) Storage modulus (solid lines) and tan δ (dashed lines) of the SMP before (dry, orange lines) and after soaking in PBS for 1 h (blue lines). Dotted line in J correlates with the values at physiological temperature, 37 °C.

We tested the ability of the MSC-16 devices to soften and wrap tightly around metal rods of several sizes. Figure 3A–D shows snug fit of the SMP substrate over rods of 100, 200, 500 and 1000 µm in diameter. This was further confirmed by implanting the MSC devices on rat peripheral nerves of different sizes, including the sensory pelvic nerve (PN) that innervates the bladder (200 µm diameter; Fig. 3E), the vagus nerve (600 µm diameter; Fig. 3F), the tibial nerve (800 µm diameter; Fig. 3G), and the ScN (1–1.5 mm diameter; Fig. 3H). In all these nerves the MSC devices conformed to their cylindrical shape, and were closed either by suture through a single eyelet and anchored onto the underlying muscle (Fig. 3E,G), or by placing a suture across two eyelets that aligned after wrapping the nerve (Fig. 3F,H). An additional closing mechanism using medical grade silicone elastomer is also illustrated in Supplementary Fig. 1. Mechanical characterization using DMA testing confirmed a 4.33 fold reduction in storage modulus (E’) of the SMP substrate, which softened from 2,380 to 550 MPa after soaking in PBS at 37 °C for 30 min (Fig. 3I). The glass transition temperature, at which the SMP substrate softens, was achieved at 45.2 °C and 66.3 °C for PBS soaked and dry electrodes, respectively (Fig. 3J). *In vitro* electrochemical measurements showed full functionality of the devices even on the smallest diameter folded cuffs (data not shown).

### Recording from somatic and autonomic nerves using the multi-size MSC-16 design

**Figure 4:**
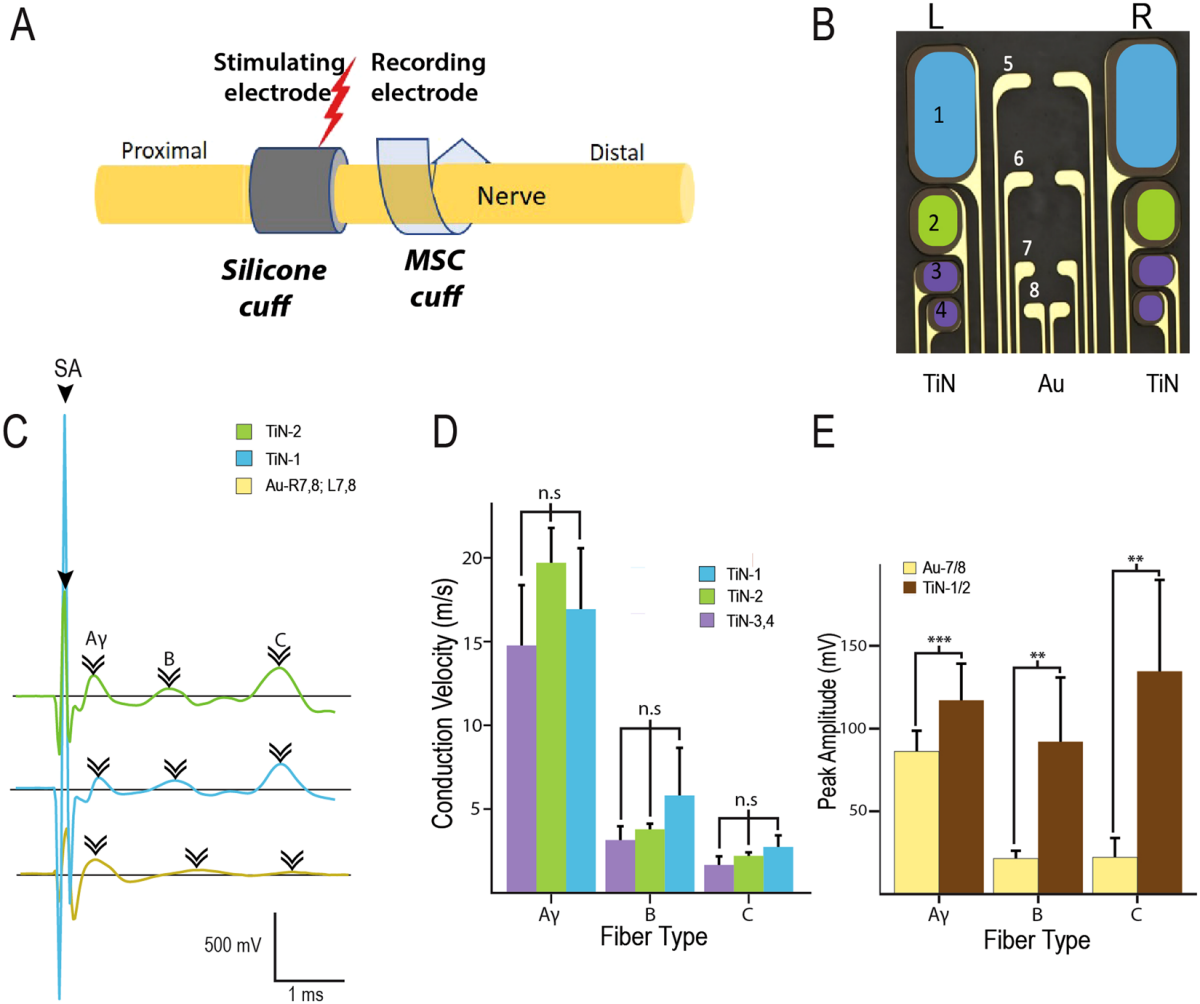
Recording activity of a somatic nerve with the MSC-16 electrode. (**A**) Setup of electrodes placed in the sciatic nerve illustrates the use of silicone cuff to evoke nerve activity and a MSC-16 cuff to record. (**B**) Diagram of the MSC-16 color-coded for electrode specific contacts represented in C-E. Aγ, **B**, and **C** fibers were identified (arrowheads in **C**) with different areas of TiN and with Au electrodes. There was reproducibility in the conductive velocity measurement using different TiN areas (**D**); with significant increase in the peak amplitude measurements at TiN contacts, compared to that at the Au electrodes (**E**). ns = non-significant; **p < 0.01, ***p < 0.001.

The acute *in viv*o functionality of the MSC-16 device was tested in two different target nerves in the adult rat: the ScN and the PN. For the larger ScN, the electrode was placed proximal to the fascicular trifurcation point of the tibial (TN), common peroneal (CPN) and sural (SN) nerves, and the CNAPs evoked by electrical stimulating using a commercial platinum/iridium (Pt/Ir) silicone cuff electrode, placed 4 mm proximal to the MSC-16 cuff (Fig. 4A). A train of depolarizing 2 mA pulses (2 Hz; 300 µs pulse duration) evoked CNAPs with Aγ, B, and C fiber components and amplitudes of 50–200 mV, recorded by the 3.3 × 10^−5^ cm^2^ Au electrodes (Fig. 4C,D). The 4.60, and 13.95 × 10^−4^ cm^2^ TiN electrodes showed higher recording sensitivity, evidenced by the detection of larger C-fiber peaks (Fig. 4C). The average conduction velocity of Aγ, B, and C peaks identified in the recorded CNAPs were similar among the TiN electrodes (Fig. 4D), and larger in average peak amplitude in the TiN-1 or TiN-2 for the Aγ: 118.02 ± 22.52 µV (p = 0.0006); B: 94.82 ± 40.09 µV (p = 0.002), and C fibers: 136.04 ± 58.56 µV (p = 0.005), compared to Au-7 + 8; Aγ: 89.64 ± 13.51 µV; B: 21.62 ± 5.41 µV; and C fibers: 22.52 ± 11.62 µV (Fig. 4E). This difference in recording sensitivity is due to the larger geometric and surface area of the TiN contacts.

**Figure 5:**
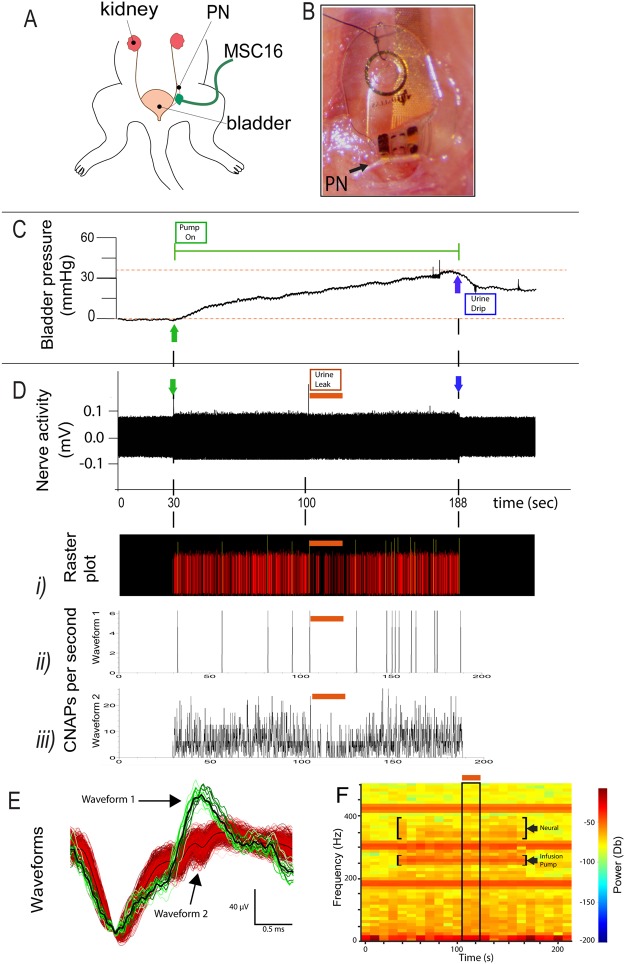
Recording mechanoceptive information from the pelvic nerve. (**A**) Illustration and (**B**) photograph of the implanted MSC-16 cuff into the sensory PN innervating the bladder. (**C**) Recorded increase in vesicular pressure during bladder filling. The green line shows the time in which the pump is “on” (green arrows), and the blue arrows indicates when the pump is off. (**D**) Neural activity recorded from the MSC-16 electrode. (*i*) Raster activity plot of two identified waveforms based on PCA analysis. Firing frequency of (*ii*) waveforms 1 and (*iii*) 2 respectively. The orange line indicates an episode of urine leak during bladder filling resulting in temporal reduction in neural activity. (**E**) Individual waveforms identified showing distinct amplitudes and patterns. (**F**) Power spectral density confirmed the identification of neural activity as it changes over the time course of bladder filling (top bracket) compared to that of the pump, which does not change over time (lower bracket). The neural activity was reduced during the urine leak episode indicated by the orange line and the boxed area.

The recording functionality of the MSC-16 cuff was also confirmed in the smaller diameter PN (Fig. 5A) during bladder filling. The electrode was secured over the 200 µm (diameter) nerve with a single suture (Fig. 5B). The root mean square (RMS) amplitude of the baseline activity was 0.05 mV. Saline infusion (770 µl total volume) increased the vesicular pressure gradually from 0 to 41.6 mmHg over 154 sec (Fig. 5C). During the increase in vesicular pressure, we recorded high frequency CNAP activity of approximately 157 µV peak-to-peak over the noise starting at 3–5 mmHg in vesicular pressure (Fig. 5D). At 112 sec of saline infusion, we noted urine leakage through the partially closed urethra, which explains the partial drop of vesicular pressure while the infusion pump is still on (horizontal orange lines in Fig. 5D). Offline sorting allowed the identification of the evoked neural activity. The raster plot in Fig. 5D-i shows the two identified waveforms, further differentiated by their distinct firing rate pattern (Fig. 5D-ii, iii), which seemed to follow the increment in vesicular pressure, and with reduced activity at the time of urine leak. The two neural waveforms are shown in Fig. 5E. Spectral density analysis of the unfiltered raw data further differentiated the neural signals from the infusion-pump noise. The neural signals increase in frequency power over time, and decrease at the time of urine leak, whereas the pump noise is constant overtime (Fig. 5F). This result demonstrated the functional use of the multi-size MSC-16 to wrap and record from nerves of different sizes.

### Fascicle-specific stimulation

**Figure 6:**
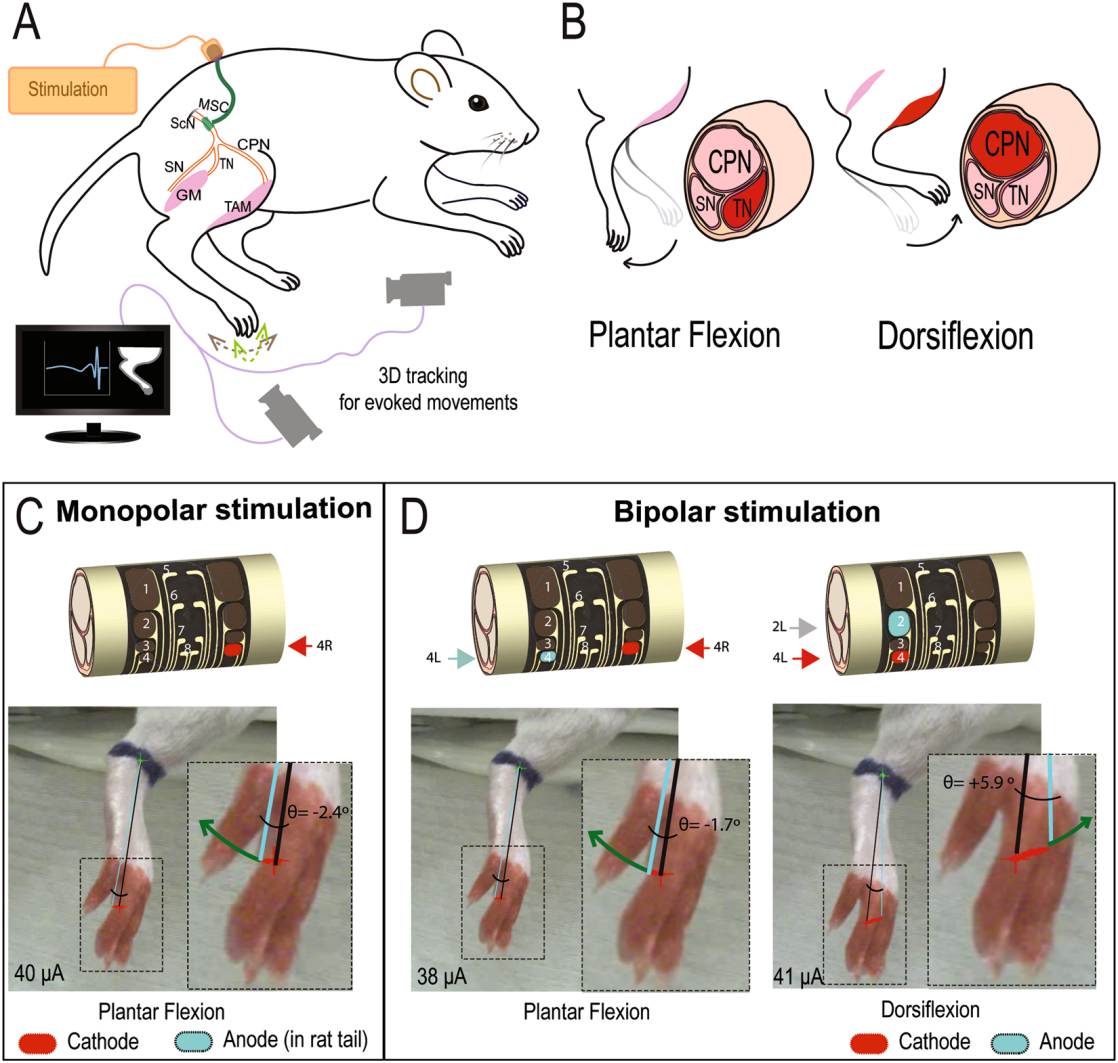
Fascicle-specific stimulation. (**A**) Experimental setup for nerve stimulation and video analysis of the evoked limb movements. (**B**) In the ScN, stimulation of the TN fascicle is known to produce plantar flexion (left), whereas selective depolarization of the CPN fascicle causes dorsiflexion (right). (**C**) Monopolar stimulation using the TiN-4R electrode evoked plantar flexion. (**D**) Bipolar stimulation with the TiN-4L/4 R electrodes induced plantar flexion, whereas the use of TiN-2L/4 L evoked dorsiflexion. ScN, sciatic nerve; SN, sural nerve; GM, gastrocnemius muscle; TAM, tibialis anterior muscle.

To demonstrate the ability of the MSC-16 cuff to evoke fascicle-specific stimulation using different electrodes on the device, we placed it over the proximal ScN. At this level, the tibial nerve (TN) and common perineal nerve (CPN) fascicles within the ScN can be differentially stimulated by steering the current, effectively contracting either the gastrocnemius muscle (GM) or the tibialis anterior muscle (TAM), respectively (Fig. 6A,B). Using similar depolarization currents (38–41 μA), monopolar (4 R) and bipolar (4R-4L) stimulation of TiN electrodes on the MSC-16 device, evoked plantar flexion (Fig. 6C,D left and Supplementary videos 1 and 2). The monopolar stimulation evoked a 30% stronger plantarflexion compared to bipolar stimulation. Conversely, bipolar stimulation using TiN electrodes 2L-4L evoked dorsiflexion, indicating selective recruitment of fibers in the CPN (Fig. 6D, right and Supplementary video 3). This result confirmed the ability of the MSC devices for localized nerve stimulation.

### Electrochemical stability of the MSC-16 after folding

Explanted electrodes were tested to evaluate the electrochemical performance of the TiN electrodes in the MSC-16 before, during, and after 27 cycles of 30 sec of *in vivo* stimulation on the ScN. The EIS showed a slight increase of impedance while implanted in the animal (Supplementary Fig. 3A,D,G). The CV spectral analysis before and after implantation on the nerve was similar, confirming that device folding does not change the electrochemical performance of the MSC-16 electrodes (Supplementary Fig. 3B,H). The radius of curvature of the implanted MSC-16 in this experiment was approximately 450 µm, and the cuff retained its folded shape upon explanting due to the shape memory property of the substrate (Supplementary Fig. 3F,I).

### Functional recording of SMP devices after 30-days *in vivo*

**Figure 7:**
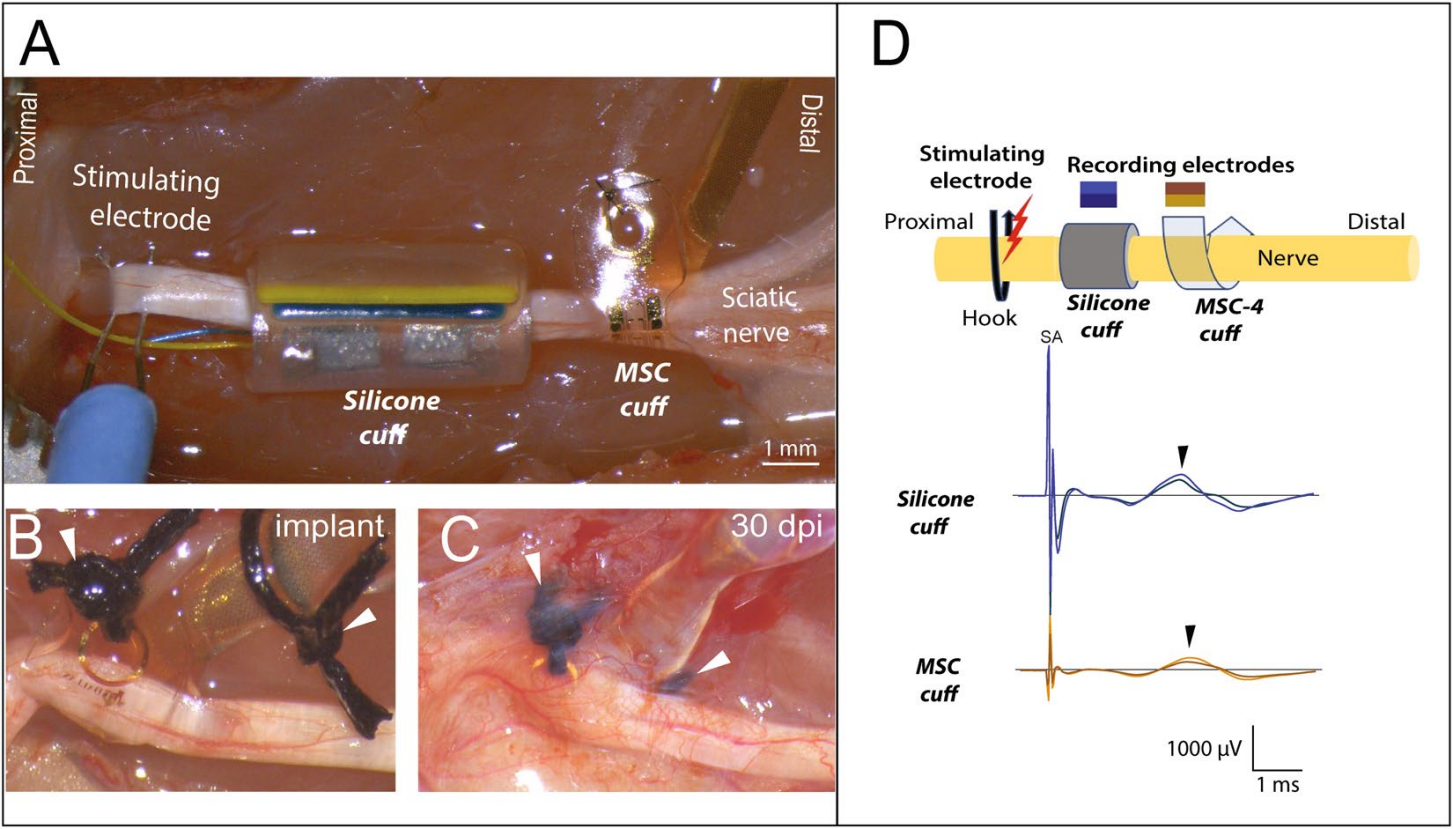
Sub-chronic recordings form the ScN. (**A**) *In vivo* setup showing the placement of the proximal stimulating hook electrode, and side-by-side implantation of the silicone and MSC cuffs electrodes. (**B**) Photograph of the SMP device immediately after placing and (**C**) 30 days after implantation, in the latter, the electrode and sutures (arrowheads) are visible through the fibrotic scar. (**D**) Schematic of the setup with color-coding of the CNAPs recorded from the silicone and MSC devices, both of which recorded the evoked Aδ fiber activity.

To test the ability of the for recording over time and directly evaluate their performance compared to conventional silicone cuff electrodes, a cohort of 9 animals was implanted with both types of cuffs onto the ScN. Thirty days post-implantation, the nerve was re-exposed and a hook electrode was placed between the sciatic notch and the silicone cuff, to evaluate the recording capability of both, the silicone cuff and the MSC-4 (Fig. 7A). After 30 days, the electrodes were re-exposed confirming that the closing/anchoring suture kept the MSC-16 cuff electrode in place (Fig. 7B,C). Gross anatomical observation upon exposure showed a thin fibrotic layer covering the SMP device, through which the sutures and electrode were clearly visible (Fig. 7C), tightly conforming to the epineurium shape as confirmed histologically (Supplementary Fig. 2). Despite the smaller size of the electrodes in the MSC-4 device (see Fig. 1C), both cuffs were able to sense Aδ fiber activation (arrowheads in Fig. 7D), as determined by the conduction velocity: 2.43 m/s in the silicone cuff and 4.04 m/s in the MSC, which are in agreement with that of myelinated pain fibers. This was confirmed by pinch stimulation of the plantar skin and silenced by lidocaine.

### Reduced fibrosis evoked by the MSC devices

**Figure 8:**
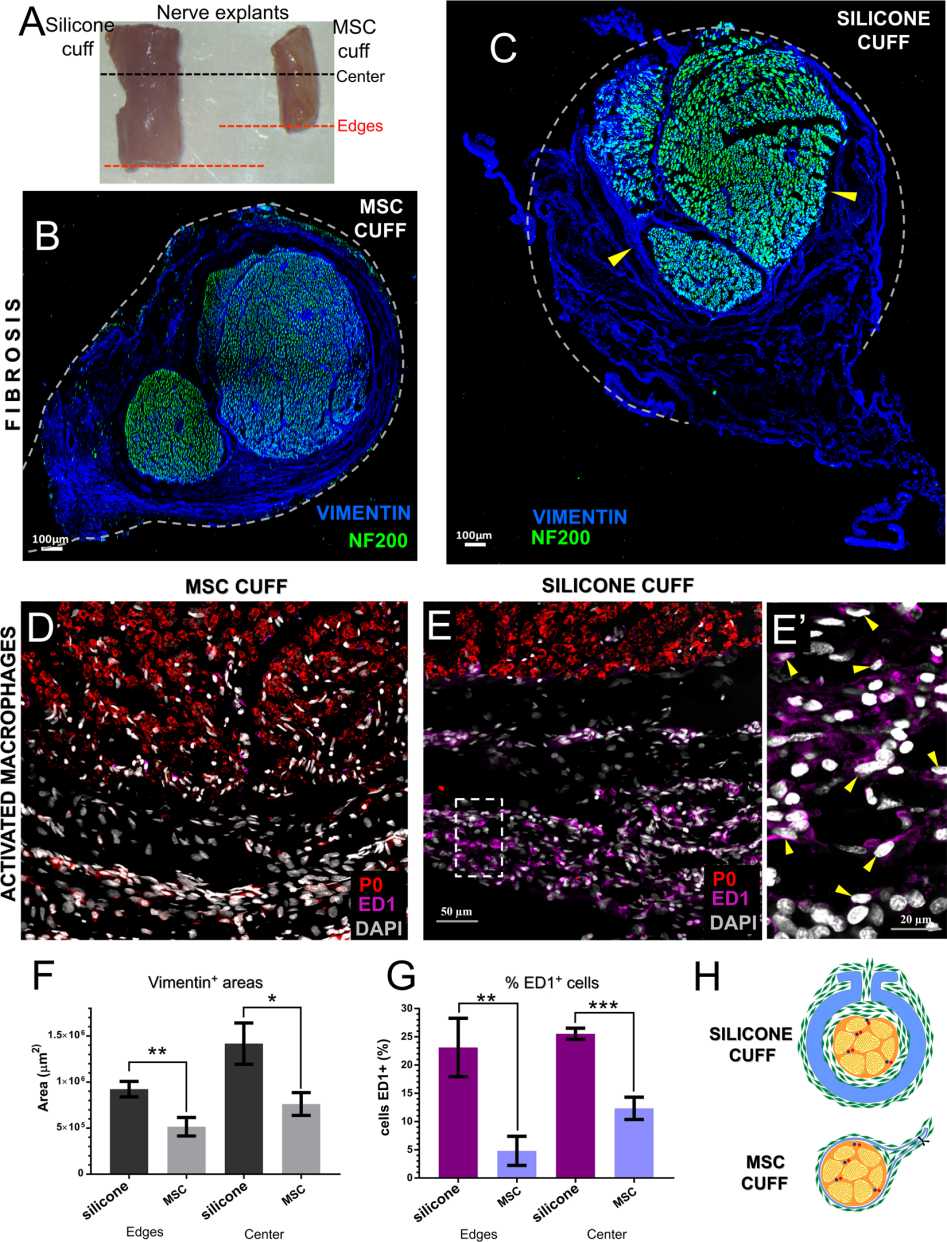
Reduced foreign body response by the SMP cuffs. (**A**) Explanted ScN 30 days after side-by-side implantation with either silicone (left) and MSC (right) electrodes. An increase in nerve diameter is evident in the segment implanted with the silicone cuff. Black and red dotted lines correspond to areas where the device center and edges were located. (**B**,**C**) Cross sections show the fibrotic tissue evidenced by vimentin labeling (blue); dotted lines indicate the relative position of the respective devices. The nerve implanted with the MSC electrode shows normal NF-200 labeled axons (green), while that implanted with the silicone cuff (**C**) shows nerve compression by fibrotic tissue ingrowth (arrowheads). (**D**,**E**) activated macrophages (ED1+ cells; pink) were increased in nerves implanted with silicone cuffs (**E**-**E**’). (**F**,**G**) The fibrotic tissue and number of activated macrophages was significantly reduced in nerve with the MSC electrode. (**H**) Schematic representation of the foreign body response. *p < 0.05, **p < 0.01, ***p < 0.001.

Gross evaluation of the explanted nerves showed that the segments with the silicone cuff were on average twice the diameter of those in the MSC electrodes (Fig. 8A). Cross sections of the nerve segments located at the middle and edges of the devices co-labeled with specific axonal (NF-200) and fibrosis (vimentin) markers, revealed diminished amount of fibrotic growth in the lumen of the MSC (Fig. 8B; Supplementary Fig. 2) compared to that of the silicone cuff. We also noted that fibrous tissue grew between the flaps of the silicone electrode and, in some animals, expanded in the lumen deleteriously compressing the nerve (arrowheads, Fig. 8C). Adjacent tissue stained with antibodies specific for myelin (P0) and activated macrophages (ED1), and counter stained with DAPI, confirmed a reduction in the number of inflammatory ED1+ cells evoked by the MSC devices (Fig. 8D). In sharp contrast, silicone electrodes increased the number of ED1+ macrophages (Fig. 8E,E’). A larger area of vimentin+ labeling was estimated on the silicone cuff implanted nerve, both at the edges (0.92 ± 0.08 mm^2^; p < 0.001) and center (1.42 ± 0.22 mm^2^; p < 0.05) of the device (Fig. 8F), compared to that of the MSC (0.51 ± 0.10 and 0.76 ± 0.12, respectively). Quantitative analysis of the activated macrophages, showed numerous ED1+ cells in the silicone cuffs similarly recruited at the edge (23.11 ± 5.18%) and center (25.55 ± 0.99%) of the devices. In sharp contrast, we observed reduction in the number of ED1+ cells in the SMP cuffs compared to the silicone electrodes (4.81 ± 2.57% at the edges, p < 0.001 and 12.34 ± 1.98% at the center, p < 0.0001; Fig. 8G). The schematic summary of the fibrotic response by each device is illustrated in Fig. 8H.

## Discussion

We report the fabrication of thin film cuff electrodes with photolithographically patterned Au and TiN contacts on a softening thiol-ene/acrylate polymeric substrate that fit snugly around peripheral nerves, allowing sensitive recording and selective stimulation with minimal fibrotic response over 30 days of implantation. Nerve cuff electrodes have been used on somatic nerves for functional electrical stimulation and brain machine interfacing of upper extremity muscles in paraplegic subjects^35,36^ or to evoke sensations in amputees^37^. These conventional cuff devices are made of soft silicone and have relative thick walls (e.g. 280–600 µm) needed to generate sufficient bending forces to keep them closed^14^, which unfortunately also causes a significant foreign body response and epineural fibrosis, negatively affecting the sensitivity of the interface^10–13^. Despite this limitation, silicone cuff devices have been shown to be effective and relatively safe when placed on somatic nerves with thousands of axons ranging 2–10 µm in diameter^38^. However, new clinical applications for the regulation of organ physiology involved in cardiac, respiratory, digestive and urinary conditions^39^, focus on neuromodulation of autonomic peripheral nerves that are smaller and composed of fewer axons (i.e., approximately 600 axons averaging 2.5 µm in the 60–80 µm rat carotid sinus nerve)^40^. The nerve targets in these conditions also have a thinner epineurium, are formed mostly of unmyelinated axons and likely more susceptible to damage by neural interface devices^39,41,42^. The small nerve size of these targets, their fragile nature, and restricted areas for implantation, are driving the development of new implantable electrodes^9^.

Elastomeric polydimethylsiloxane microchannel devices have shown to be effective in recording pelvic nerve activity, but this strategy requires teasing the nerve and placing the small nerve bundles inside a 100 µm microchannel^14,43^. We have previously reported a silicon microchannel electrode-array and validated it in acute studies for the fascicular stimulation of the rat deep peroneal nerve^44,45^, but the device is stiff and was not designed for chronic implantation. Alternatively, cuff electrodes have been fabricated using polyimide thin film technology (10–25 µm), and demonstrated to be effective in interfacing peripheral nerves without causing compression injury^19^. However, polyimide is stiffer (3 GPa) than silicone (1 MPa)^22^, the cuffs are fabricated for pre-determined sizes^19^, and some need to be rolled inside a silicone tube for *in vivo* testing^14^.

This study demonstrates the effectiveness of the thiol-ene/acrylate polymeric substrate for the fabrication of a multi-size 16-contact cuff device with Au and TiN electrodes. The four-row layout of the MSC-16 cuff was designed to fit nerves ranging from 100 to 1000 µm diameter, and is capable of bending over a 100 µm radius without compromising the electrical performance of the device. The MSC-16 has eyelets that can be used for handling, and to place a closing suture by anchoring the folded segment of the electrode to the underlying muscle. The MSC-12 design has eyelets that can be aligned and closed by suture after rolling the film into a cuff. We confirmed that the MSC-16 cuff is able to wrap around the 200 µm diameter PN and record neural activity evoked by bladder filling, where two specific wave forms with distinct activity patterns were identified. The waveforms likely represent the two populations of Aδ mechanoceptors known to innervate the bladder, and responsive to pressure changes^46,47^. Since the same electrode can be placed on the ScN (1000 µm diameter) to record CNAPs with Aγ, B, and C fiber components, it confirmed the ability for these softening cuff electrodes for neural recording of multiple nerve sizes.

While some reports provide controversial evidence regarding the charge injection limit of TiN (0.87 mC/cm^2^) compared to iridium oxide (IrO) (4 mC/cm^2^)^29^, others have shown that this material provides sufficient charge injection (4.45 mC/cm^2^) within voltage and current safety limits^30^. In this study, the 220 nm TiN film formed a pyramidal microstructured coating on the Au electrodes significantly increasing the total surface area, providing a CSC of 3 mC/cm^2^, closely resembling that of IrO.

Multi-contact cuff electrodes are used for selective fascicular activation based on the geometric configuration of the contacts^48–50^. The multiple contacts of the MSC-16 cuff allowed selective nerve stimulation from the ScN, as bipolar activation of the same raw electrodes such as TiN-4 and TiN-8 in the MSC-16 device, recruited the TN fascicles and evoked plantar flexion. Conversely, activation of the TiN-3 and TiN-4 contacts, resulted in selective recruitment of motor axons in the CPN fascicle, evoking dorsiflexion paw movements. This selective fascicular stimulation is similar to that reported previously with multi-electrode polyimide cuffs^51^, and could have specific clinical applications. It will be interesting to test if different combinations of electrode contacts in the vagus nerve can be used to avoid unwanted arrhythmias and laryngopharyngeal dysfunction, commonly attributed to the recruitment of off-target nerve fascicles^52,53^. Notably, we were able to recruit fascicular activation with relatively low currents from both Au and TiN electrodes (e.g., 30–40 µA), an order of magnitude smaller compared to the polyimide cuff and similar to intra-fascicular electrodes^51^.

A side-by-side comparison of the silicone cuff and the MSCs 30 days after implantation showed that both devices were able to record CNAP activity. The smaller contact size in the MSCs was compensated by the increase in surface area by TiN. Similarly, both electrodes were equally effective in recording nerve activity a month after implantation. However, the main difference between the two types of electrodes was noted when the nerve segments implanted with the respective devices were evaluated histologically. Labeling of the fibrotic tissue growth by vimentin showed that the fibroblasts migrated into the lumen of the silicone cuff and in some cases compressed the nerve. This was not observed in any of the nerves implanted with the SMP cuffs. We also noted that the fibrotic activity in the silicone cuffs was active after 30 days, as indicated by the visualization of a significant number of ED1+ activated macrophages. This result suggested a continued insult to the nerve by the thick silicone device, likely involving the release of tumor necrosis factor-alpha and transforming growth factor-beta1 as previously reported for these types of electrodes, and that has been linked to partial nerve injury^54,55^. In sharp contrast, the number of ED1+ cells in the nerve segment implanted with the MSC cuffs was significantly lower, indicating less damage to the nerve. This can be the result of less bulk foreign material by the use of thin film electrodes and the softening properties of the thiol-ene/acrylate polymeric substrate.

The reduced fibrotic response by the MSCs is likely explained by a combination of the softening nature of the SMP substrate and the thin film manufacturing, which allows for a snug fit on the nerve. Our DMA results indicate that the SMP used in this study softens to approximately 550 MPa, roughly an order of magnitude higher of our previous report^56^. This is due to the extended photo-polymerization process we implemented for the devices used in this study, which results in higher cross-link density and a slightly shifted glass transition temperature. At physiological conditions, the SMP substrate is expected to be in its viscoelastic state (between glassy state and rubbery) and able to accommodate significant forces without failure. While the SMP material is less flexible compared to silicone, it allows the fabrication of thin-film devices, resulting in 30 µm thick cuffs with 300 nm layers of Au/TiN. These devices are 20-fold thinner compared to commercial silicone cuffs that have 200–600 µm thick walls, and micron-sized Pt or IrO wire electrodes. The thick walls of the silicone cuffs and the larger amount of metallic material significantly increase the hoop stress of the cuff, and consequently, its elastic modulus approximately 10–100 fold higher compared to the SMP cuffs. Indeed, when the full devices were tested, flexural forces were about 70–700 times lower in the SMP cuffs. Thus, the use of the SMP as a substrate material and a thin-film fabrication methods, resulted in MSC that are more compliant with the nerve tissue. Moreover, a suite of thiol-ene/acrylate based SMP substrates with various level of softening have been previously demonstrated, and we have also shown that the SMP material maintains its thermo-mechanical properties after ethylene oxide sterilization^57^. The electrochemical characteristics of the MSC electrodes after implantation showed that these devices maintained their performance after removal, further supporting their robustness in acute testing. However, while these results demonstrate the use of MSC for acute and sub-chronic studies, evaluation of this softening cuff electrodes in chronic studies are needed in order to confirm the functionality of these devices over long periods of time, conditions that would more closely resemble potential clinical applications.

In summary, we report the fabrication and characterization of thiol-ene/acrylate multi-contact cuff electrodes that soften at body temperature and can be snugly wrapped around nerves of different sizes, providing optimal nerve-electrode contact with reduced foreign body response. These unique characteristics make the MSC electrodes a viable alternative for peripheral nerve interfaces for sensitive recording and safe electrical stimulation of small and fragile nerves, thus enabling access to a number of autonomic nerve targets for clinical neuromodulation applications.

## Electronic supplementary material

Supplementary Information

Supplementary Video 1. Monopolar selective stimulation evoking hind limb plantar flexion.

Supplementary Video 2. Bipolar selective stimulation evoking hind limb plantar flexion.

Supplementary Video 3. Bipolar selective stimulation evoking hind limb dorsi flexion.
