# Supplementary material for: Thin Film Multi-Electrode Softening Cuffs for Selective Neuromodulation
Source: Sci Rep. 2018 Nov 6;8:16390. doi: 10.1038/s41598-018-34566-6 (PMC6219541; doi:10.1038/s41598-018-34566-6)
Supplement: Supplementary file 1 — Supplementary Information [file 41598_2018_34566_MOESM1_ESM.pdf]

Thin Film Multi-Electrode Softening Cuffs for Selective Neuromodulation

María Alejandra González-González[1], Aswini Kanneganti[1], Alexandra Joshi-Imre[2], Ana Guadalupe Hernandez-Reynoso[1], Geetanjali Bendale[1], Romil Modi[2], Melanie Ecker[2], Ali Khurram[2], Stuart F. Cogan[1], Walter E. Voit and[2], Mario Romero-Ortega[1*]

[1] Departments of Bioengineering and [2]Material Science and Engineering, University of Texas at Dallas, 800 W. Campbell Road, Richardson, TX, 75080.

*Corresponding author: Mario I Romero-Ortega, PhD. Mario.Romero-Ortega@utdallas.edu

Supplementary Information

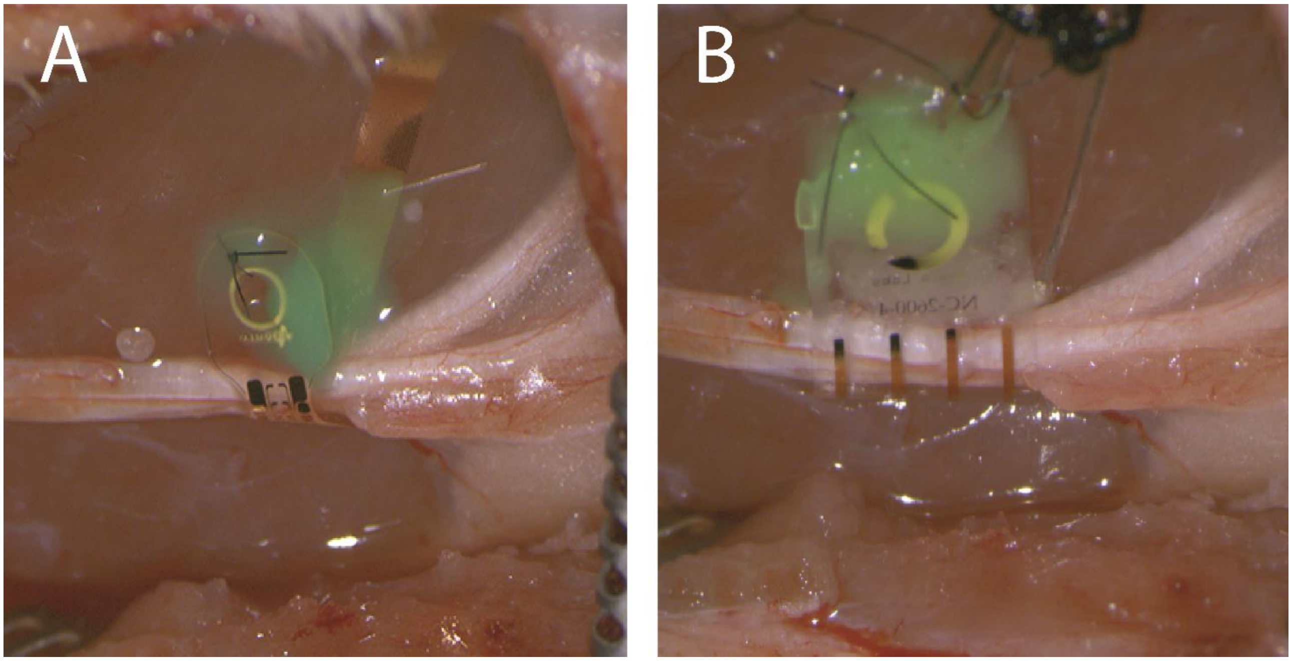

Supplemental Figure 1. Closing mechanism of MSC cuffs using biocompatible silicone elastomer. Sciatic nerve implanted with MSC-16 (A) and MSC-4 (B), using sutures and/or a biocompatible silicone elastomer as closing mechanism.

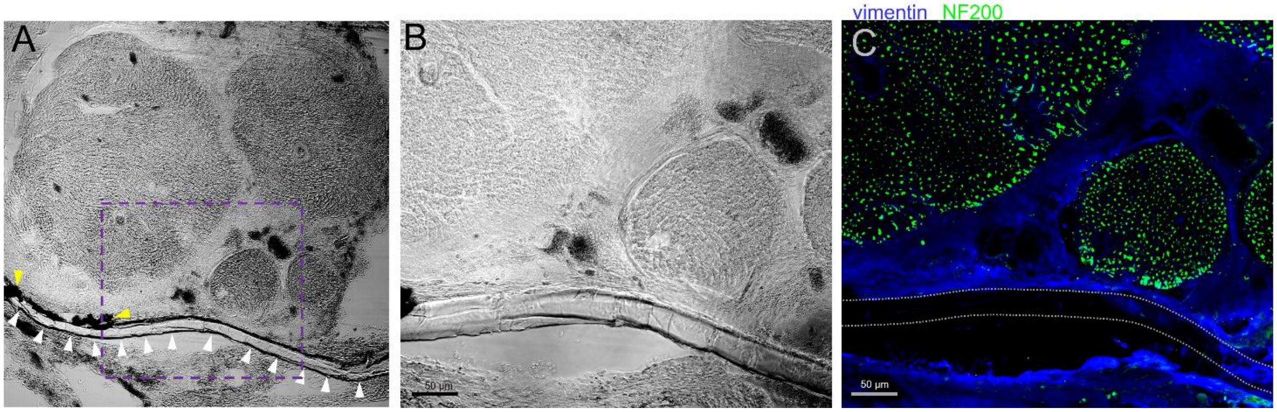

Supplemental Figure 2. MSC cuff adapts to the nerve epineurium shape. (A-B) Two magnifications of DIC images from the sciatic nerve (B represents the dotted square in A) after 30 days implanted with a MSC cuff (white arrowheads). The electrode traces after tissue section (yellow arrowheads) evidence preserved nerve tissue underneath. (C) Vimentin and NF200 immunostainings were used to contrast the epi and endoneurium and the nerve tissue, MSC cuff is delineated with dotted lines.

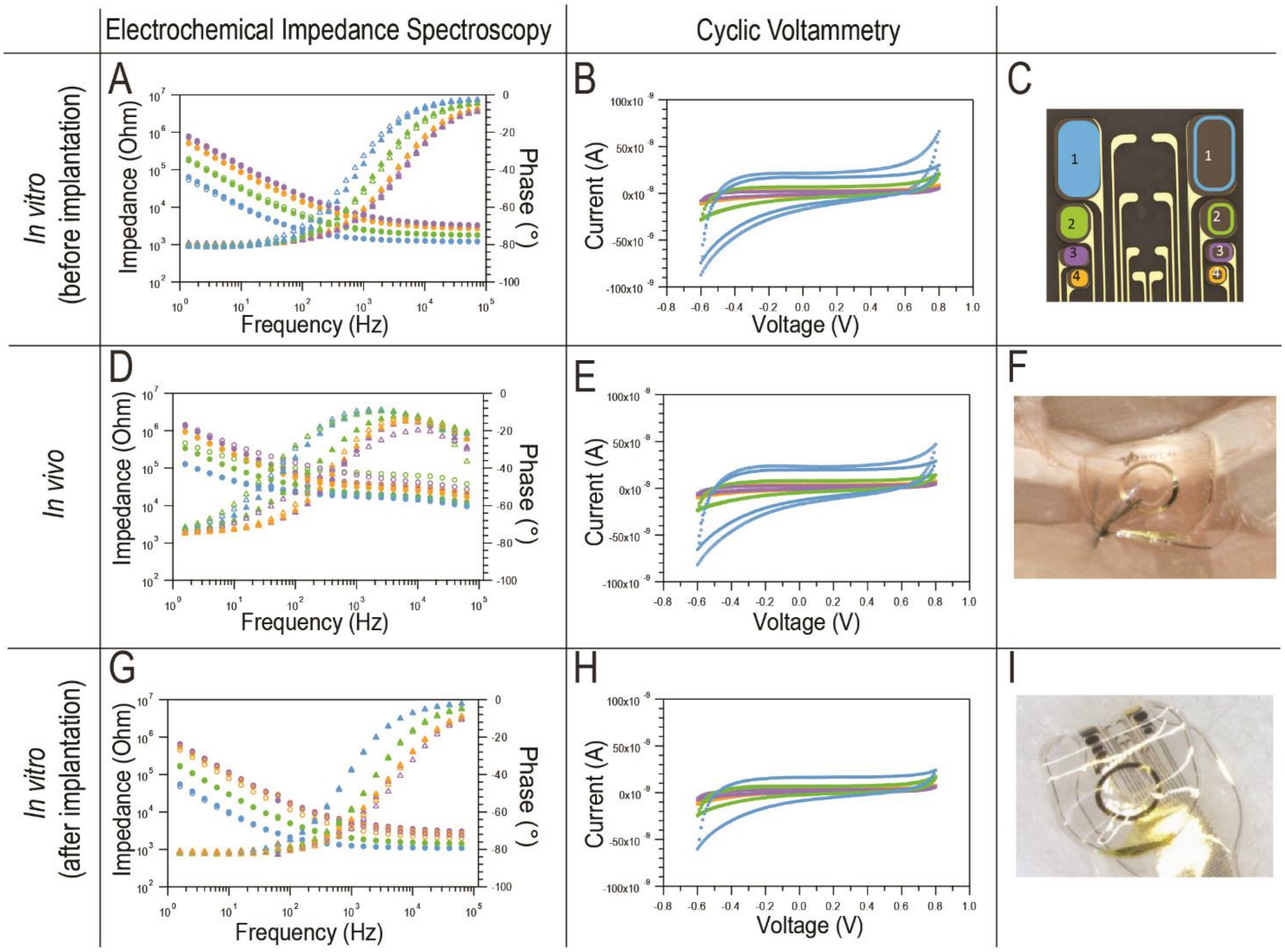

Supplemental Figure 3. Electrochemical properties of the MSC-16 after acute implantation. Electrochemical evaluation of TiN electrodes before (A, B), during (D, E) and after (G, H) implantation into the ScN. A, D and G show the potentiostatic EIS values (circles represent the impedance, triangles the phase), whereas B, E and H show the respective CV measurements. C displays the legend for color-coding for the electrochemical measurements. Filled symbols represent the electrodes on the left side of the cuff, while the open symbols represent values of the symmetrical electrodes on the right side. F and I show pictures of the device *in vivo* and explanted, respectively.

Supplemental Video 1. Monopolar selective stimulation evoking hind limb plantar flexion. Monopolar stimulation with the smallest TiN electrode (red in the schematic bottom right) evokes hind limb plantar flexion. Experimental parameters are displayed in the bottom left.

Supplemental Video 2. Bipolar selective stimulation evoking hind limb plantar flexion. Bipolar stimulation using specific TiN electrode combinations (cathode in red and anode in blue in the schematic bottom right) evokes hind limb plantar flexion. Experimental parameters are displayed in the bottom left.

Supplemental Video 3. Bipolar selective stimulation evoking hind limb dorsi flexion. Bipolar stimulation using specific TiN electrode combinations (cathode in red and anode in blue in the schematic bottom right) evokes hind limb dorsi flexion. Experimental parameters are displayed in the bottom left.
